# Effect assessment of laparoscopy in combination with traditional Chinese medicine decoction in the treatment of endometriosis: A protocol for systematic review and meta-analysis

**DOI:** 10.1097/MD.0000000000026699

**Published:** 2021-07-23

**Authors:** Jiahua Peng, Ruiqi Wang, Zhiling Ding, Xin Song

**Affiliations:** a Institute of Obstetrics and Gynecology of Traditional Chinese Medicine, Jiangxi University of Chinese Medicine, Nanchang, Jiangxi Province, China; b Jiangxi University of Chinese Medicine, Nanchang, Jiangxi Province, China; c College of Traditional Chinese Medicine, Jiangxi University of Chinese Medicine, Nanchang, Jiangxi Province, China.

**Keywords:** endometriosis, laparoscopy, meta-analysis, traditional Chinese medicine

## Abstract

**Background:**

Endometriosis (EMs) affects about 10% of women of childbearing age. It is defined as functional endometrial tissue appearing in other parts of the uterine cavity, manifested by varying degrees of pelvic pain and pelvic mass, etc. Therefore, to improve the therapeutic effect of endometriosis, we must constantly explore new ways to treat the disease. The purpose of this study is to evaluate the effectiveness and safety of the combined use of laparoscopy and traditional Chinese medicine in the treatment of patients with EMs.

**Methods:**

A systematic literature search will be conducted at China National Knowledge Infrastructure, WanFang databases, VIP, SinoMed, PubMed, Embase, Web of Science, and the Cochrane library. The search period limit is from the time the date of database establishment to June 21, 2021. To ensure the comprehensiveness of the search, relevant references and conference literature are also included. The risk of bias in the final included studies will be evaluated based on the guidelines of the Cochrane Handbook for Systematic Reviews of Interventions. The RevMan software will be employed to perform data synthesis and statistical analysis.

**Results:**

The effectiveness and safety of laparoscopic surgery combined with traditional Chinese medicine decoction in the treatment of patients with EMs will be systematically evaluated.

**Conclusion:**

The results of this study will provide strong evidence for judging whether laparoscopy combined with traditional Chinese medicine decoction is an effective strategy for the treatment of patients with EMs.

## 1 Introduction

Endometriosis (EMs), an estrogen-dependent inflammatory disease, is one of the most common chronic gynecological disorders affecting women in reproductive age. Its histological characteristics are that the endometrial glands and stroma appear outside the uterine cavity, grow, infiltrate, repeatedly bleed and form nodules or masses, which can cause extensive and serious adhesions.^[1]^ EMs appears to be one of the most common benign gynecological proliferations in premenopausal women since it is estimated that 10% to 15% of reproductive aged women suffer from pelvic endometriosis, and the incidence in women suffering from chronic pelvic pain and infertility, is as high as 30% to 60%.^[2,3]^ However, the pathogenesis of EMs is still unclear, the diagnosis is inaccurate, and the unsatisfactory treatment is a well-known “refractory disease” among gynecological diseases.^[4]^ The main clinical manifestations of EMs are chronic pelvic pain that seriously affects women's normal quality of life, secondary and progressive dysmenorrhea, infertility, and sexual discomfort.

The world congress on endometriosis concluded that the best treatment for endometriosis is: laparoscopy, ovarian suppression, “three-phase therapy,” pregnancy, assisted pregnancy.^[5]^ In recent years, laparoscopy has gradually replaced open surgery with its advantages of minimal invasiveness, fewer complications, and fast healing, but surgical treatment alone still cannot solve the problem. EMs are called “pelvic sandstorms,” both radical and conservative operations can only achieve the goal of reducing the removal of visible lesions and restoring normal anatomical structures as much as possible. The recurrence rate of postoperative pain or endometriosis of the ovary remains high. In traditional Chinese medicine, the corresponding disease names are “dysmenorrhea,” “infertility,” and so on. There are a large number of domestic literatures that Chinese medicine can effectively treat EMs. The treatment of endometriosis with traditional Chinese medicine plays an effective clinical role in alleviating the pain of the disease, slowing the progression of the disease, and preventing the recurrence of the disease after the operation, and has clinical advantages.^[6]^

Modern studies have shown that integrated traditional Chinese and Western medicine has a good clinical effect in the treatment of EMs. However, the existing domestic and foreign literatures on the treatment of EMs with integrated traditional Chinese and Western medicine are numerous and messy, lacking of systematic reviews, and meta-analysis results in insufficient clinical evidence for the treatment of dysmenorrhea by laparoscopy and traditional Chinese medicine. Through systematic evaluation of the clinical efficacy of laparoscopy combined with traditional Chinese medicine in the treatment of EMs, to guide Chinese and Western doctors in the rational treatment of EMs and relieve the pain of dysmenorrhea patients. To obtain conclusive evidence of the efficacy of laparoscopy combined with traditional Chinese medicine in the treatment of EMs. This study uses evidence-based medicine to find clinical randomized controlled trials (RCTs) for the application of laparoscopy in combination with traditional Chinese medicine decoction in the treatment of EMs to provide evidence-based evidence for clinicians.

## 2 Methods

The study is conducted according to the Preferred Reporting Items for Systematic Reviews and Meta-Analyses Protocols (PRISMA-P) statement. The protocol of the systematic review has been registered in the INPLASY website (registration number is INPLASY202160074).

### 2.1 Eligibility criteria of inclusion of studies

#### 2.1.1 Types of studies

We will include all RCTs investigating laparoscopic surgery combined with Chinese herbal decoction in the treatment of EMs. It will be excluded if the following situations are included.

1. The outcome indicators in the literature are not clear or the outcome indicators cannot be extracted.
2. For research and reports with repeated substantive content from the same unit or the same time period and signed by the same author, select one of them as the target document.
3. Interventions incompatible with the inclusion criteria and animal experiments.

#### 2.1.2 Types of participants

Patients diagnosed with EMs, the diagnosis meets the guidelines of the American Society of Reproductive Medicine ^[7]^ diagnostic criteria for endometriosis. There will also be no restrictions based on gender, race, and the course of the disease. However, the EMs combined with pelvic infections, endometrial polyps, intrauterine adhesions, cervical stenosis, uterine malformations, and other diseases will be excluded.

#### 2.1.3 Types of interventions

The experimental group was laparoscopic surgery combined with Chinese medicine decoction, and the control group was simply laparoscopic surgery.

#### 2.1.4 Types of outcome measures

The primary outcome indicators include serum follicle-stimulating hormone, luteinizing hormone, estradiol, pregnancy rate, recurrence rate. The minor outcomes include: An-tral follicles count, ovarian volume, and adverse reactions.

### 2.2 Electronic searches

A systematic literature search will be conducted at China National Knowledge Infrastructure, WanFang databases, VIP, SinoMed, PubMed, Embase, Web of Science, and the Cochrane library. The search period limit is from the time the date of database establishment to June 21, 2021. To ensure the comprehensiveness of the search, relevant references and conference literature are also included. The search will identify all randomized controlled trials to evaluate the effectiveness and safety of laparoscopy combined with Chinese herbal decoction in the treatment of EMs. Afterward, the Boolean operators “OR” and “AND” will be used to combine the following search terms: “Ems,” “Endometriosis,” “Chinese medicine decoction,” “traditional Chinese medicine,” “TCM,” “randomized controlled trial,” “RCT.”

### 2.3 Data collection and analysis

#### 2.3.1 Selection of studies

**Figure 1:**
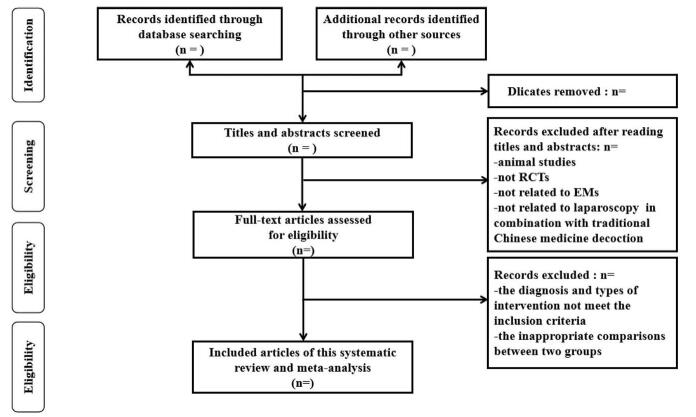
The research flowchart.

According to the stipulated Chinese and English search terms, the aforementioned databases were searched separately. Two reviewers strictly followed the established inclusion and exclusion criteria to initially screen the relevant documents, read the titles and abstracts of the documents, and preliminarily screened the documents, and rejected. For the documents that meet the requirements, carefully read the full text of the documents that may be included. If there is a difference in the selection of the documents, the discussion or the assistance of a third researcher will be used to determine. For experiments with similar contents of the literature, carefully check the title, author, unit, number of samples, observation indicators, and other characteristics to determine whether it is a duplicate of the literature. If it is judged to be a duplicate, only one of them will be included in the study. The flowchart will be demonstrated in Figure 1.

#### 2.3.2 Data extraction and management

After the documents passed the screening, 2 researchers used the designed electronic form to extract the data and data from the included documents, and if they disagree, the third researcher would determine if they disagree. Documents lacking detailed data should be improved by contacting the author via email. If the author fails to reply, it will be treated as invalid.

#### 2.3.3 Assessment of risk bias

The risk of bias in the final included studies will be evaluated based on the guidelines of the Cochrane Handbook for Systematic Reviews of Interventions. The evaluation criteria include 7 items: selections bias, performance bias, detect bias, attrition bias, reporting bias, and other bias. Each item will be graded into 3 levels: “high risk,” “low risk,” and “not clear.”^[8]^ This work will also be done independently by 2 reviewers.

#### 2.3.4 Measures of treatment effect

Revman 5.3 was used to perform statistical analysis on the extracted data and draw forest maps. Choose appropriate statistics and statistical methods according to the type of data. For binary data, calculate its odds ratio and its 95% CI. For measurement data, calculate the weighted mean difference and 95% CI.

#### 2.3.5 Assessment of heterogeneity

The *χ*^2^ test is used to test the heterogeneity of the included literature, and the test level is α = 0.10, and *I*^*2*^ is used to quantitatively analyze the heterogeneity.^[9]^ When the heterogeneity of the results of a study is small (*P* > .1, *I*^2^ < 50%), a fixed-effect model is selected for statistical analysis.

#### 2.3.6 Assessment of sensitivity analysis

If the heterogeneity between the research results is large (*P* ≤ .1, *I*^2^ ≥ 50%), sensitivity analysis should be used to explore the source of the heterogeneity. The main methods of sensitivity analysis are: change the analysis model, when the heterogeneity is high, use the random effects model. Documents are excluded one by one. If the heterogeneity does not change after being eliminated separately, the result is relatively robust. If the heterogeneity of a certain document is eliminated, then this article may be the source of the heterogeneity. According to the different characteristics of the literature, such as region, dose, etc, conduct subgroup analysis.

#### 2.3.7 Assessment of reporting biases

If the outcome indicators include in study ≥10, funnel plots will be used to assess the publication bias of the included trials.^[10]^ If there is a difference in symmetry or distribution, there will be a publication bias or a small sample effect.

### 2.4 Ethics and dissemination

Because this is a protocol for systematic review and network meta-analysis, all data of this study are from published studies and do not involve patients, so ethical approval will not be necessary. The findings of this study will be disseminated to a peer-reviewed journal and presented at a relevant conference.

## 3 Discussion

When endometrial tissue with growth function appears in the uterine cavity covering membrane and other parts of the uterine body, it is called EMs. Secondary progressive dysmenorrhea, chronic pelvic pain, infertility, and pelvic mass are the main clinical manifestations. The incidence rate has been increasing in recent years, and 30% to 50% of patients with endometriosis are often accompanied by infertility.^[11]^ Laparoscopy is currently the best method for diagnosis of EMs, and it is also the best treatment method. At present, laparoscopic diagnosis, surgery + drug treatment are the gold standard for the treatment of endometriosis.^[12]^ Western medicine uses hormonal drug treatment and assisted pregnancy technology to obtain a certain clinical pregnancy rate, but due to more adverse reactions, more and more patients choose to seek traditional Chinese medicine for treatment. In clinical research, a randomized controlled study (RCT) is the clinical research that evaluates the best efficacy of therapeutic interventions. The Cochrane system evaluation based on RCT is the gold standard for evaluating interventions and is recognized as one of the most reliable evidences for evaluating clinical efficacy. In recent years, the number of RCTs in the treatment of EMs by laparoscopy combined with Chinese medicine decoction has gradually increased, but there is a lack of systematic reviews and meta-analysis of scientific research methods. At present, the systematic evaluation of the clinical efficacy of Chinese patent medicines in the treatment of secondary dysmenorrhea EMs is still blank. This study will systematically evaluate the RCT of laparoscopy combined with traditional Chinese medicine decoction in the treatment of EMs published at home and abroad in recent years, and conduct meta-analysis for the research with small heterogeneity, so as to provide the effectiveness and safety provides evidence-based medicine.

## Author contributions

**Conceptualization:** Jiahua Peng, Ruiqi Wang, Zhiling Ding, Xin Song.

**Data curation:** Jiahua Peng, Ruiqi Wang, Zhiling Ding.

**Formal analysis:** Jiahua Peng, Zhiling Ding, Xin Song, Ruiqi Wang.

**Funding acquisition:** Jiahua Peng, Ruiqi Wang, Xin Song.

**Investigation:** Ruiqi Wang, Zhiling Ding.

**Methodology:** Jiahua Peng, Ruiqi Wang, Xin Song.

**Resources:** Jiahua Peng, Zhiling Ding, Xin Song.

**Software:** Ruiqi Wang, Zhiling Ding, Xin Song.

**Supervision:** Jiahua Peng, Ruiqi Wang, Xin Song.

**Validation:** Ruiqi Wang, Zhiling Ding, Xin Song.

**Visualization:** Jiahua Peng, Xin Song.

**Writing – original draft:** Jiahua Peng, Ruiqi Wang, Xin Song.

**Writing – review & editing:** Jiahua Peng, Zhiling Ding.
